# Self-Efficacy of People with Chronic Conditions: A Qualitative Directed Content Analysis

**DOI:** 10.3390/jcm7110411

**Published:** 2018-11-03

**Authors:** Fatemeh Ebrahimi Belil, Fatemeh Alhani, Abbas Ebadi, Anooshirvan Kazemnejad

**Affiliations:** 1 Nursing Department, Tarbiat Modares University Tehran, Tehran 14115-331, Iran; alhani_f@modares.ac.ir; 2 Behavioral Sciences Research Center, Life style institute, faculty of nursing, Baqiyatallah University of Medical Sciences, Teheran, Iran; Ebadi1347@bmsu.ac.ir; 3 Biostatistics Department, Tarbiat Modares University, Tehran 14115-331, Iran; alhani_f@modares.ac.ir

**Keywords:** chronic disease, self-efficacy, power, caregivers, empowerment

## Abstract

Background: Given the increasing prevalence of chronic illnesses and their complications, supporting and empowering chronically ill patients seems crucial. Self-efficacy is considered as a predictor for empowerment. The purpose of this study to explore of different aspects of self-efficacy among persons with chronic physical conditions based on the Family-Centered Empowerment Model (FCEM). Methods: this qualitative study is part of a larger study; sequential exploratory mixed-method for designing an instrument for the FCEM was conducted from May 2015 to March 2016 in two university hospitals. The sample was 22 participants, including chronically ill patients, family caregivers, and nurses. Data were collected through personal semi-structured interviews. Data analysis was performed concurrently with data collection through directed qualitative content analysis. Results: after determining the self-efficacy attributes in the family-center empowerment model, a category matrix was developed and the codes are placed in subcategories of the matrix. Most participants were female (58.0%), with a mean age of 49.50 years. The final analysis yielded a total of 247 units of analysis dispersed in eight subcategories belonging to four generic-categories. Conclusions: the findings of this study represent the dimensions of chronically-ill individuals’ self-efficacy that can be used to develop and implement programs for empowering chronic ill patients.

## 1. Introduction

Currently, more people are living with debilitating chronic conditions (such as cardiovascular, diabetes, asthma, rheumatism, and blood illness). These conditions and their management are the most challenging life events and global health problems [1]. Due to their chronic nature, these conditions cause different psychosocial problems, endanger patients’ identities, change their roles and lifestyles [2], and make them unable to fulfill their needs [3]. Therefore, patients with chronic diseases need extensive care, support, and empowerment-based services in order to cope with their diseases. By definition, empowerment is “the process that helps people gain control over their own lives and increases their capacity to act on issues that they themselves define as important” [4]. It is associated with greater confidence, autonomy, self-efficacy, and self-determination in doing activities of daily living as well as self-care activities [4]. It enhances quality of life, strengthens accountability and patient interactions with healthcare providers, promotes treatment response, reduces healthcare costs, helps prevent complications, and increases self-confidence and control over life [3].

Self-Management is defined as “the ability of the individual, in conjunction with family, community, and healthcare professionals, to manage symptoms, treatments, lifestyle changes, and psychosocial, cultural, and spiritual consequences of health conditions” and self-care is defined as “deliberately performed actions to regulate human functioning and development” [5].

Empowerment results in satisfaction, self-efficacy, self-esteem, life expectancy, hope, quality of life, decision-making, education, and counseling, and it has different levels and models [6].

The term was first introduced by Albert Bandura and is defined as someone’s belief in their own ability to successfully perform an activity and achieve the expected outcomes. Studies on empowerment show that self-efficacy is significantly correlated with empowerment [7]. Self-efficacy is also considered an important concept in the assessment and improvement of chronic conditions (self-management, quality of life, behavioral modification, hopefulness, lifestyle modification, physical and mental health, and disease prevention) [8,9].

One of the most comprehensive and applicable models for family empowerment is the Family-Centered Empowerment Model (FCEM). It is a context-based comprehensive model which was developed through an original grounded theory study [4]. It cannot be denied that the form of family is changing in today’s world, and there are many forms of family. However, in the Muslim community of Iran, the majority form of families is nuclear and secondarily widespread, and other forms of the family are not formal. In spite of the transition period of the family concept, the relationships in the family still exist with the core of the family, which creates and maintains a supportive relationship between the children and parents [10]. The model has four stages: (1) perceived threat (group discussion method); (2) self-efficacy (problem-solving method); (3) self-esteem (educational participation method); and (4) process and outcome evaluation [4]. The FCEM highlights the important roles of individuals and their family members in promoting well-being and quality of health. The main goal of the model is to empower the family system (patients and their main family caregivers) to promote their own health [4].

Previous studies also confirmed the positive effects of the FCEM on self-confidence, goal attainment ability, control over life and change-related processes, hopefulness about the future, self-care and self-efficacy, and quality of life [11]. One of the key concepts of the FCEM, and the most important acquired skill for empowerment, is self-efficacy. In this model, using the skill acquisition method, the patient’s self-efficacy comes first and then, through educational participation, improvement of the family’s empowerment [7].

The term was first introduced by Albert Bandura and is defined as someone’s belief in their own ability to successfully perform an activity and achieve the expected outcomes. Studies on empowerment show that self-efficacy is significantly correlated with empowerment [7]. Self-efficacy is also considered an important concept in the assessment and improvement of chronic conditions (self-management, quality of life, behavioral modification, hopefulness, lifestyle modification, physical and mental health, and disease prevention) [8,9].

Therefore, studies and programs for enhancing individuals’ self-efficacy based on their own conditions, abilities, and needs are among the top healthcare priorities of families. Nonetheless, to our knowledge, there is limited information about chronically ill peoples’ perceptions of their self-efficacy. Consequently, the present study was made to explore the different aspects of self-efficacy among patients with chronic conditions based on the FCEM.

## 2. Materials and Methods

### 2.1. Study Design

Qualitative methods are ideal for collecting detailed data and creating deep understanding about the phenomena and concepts of interest [12]. This qualitative study is part of a larger study sequential exploratory mixed-method for designing an instrument for the FCEM. Researchers use this design when existing instruments, variables, and measures may not be known or available for the population under study [13]. Sometimes, there are existing theories or prior research about a phenomenon that are incomplete or would benefit from further description or test of the similarity of meaning of concept in a different setting. A deductive approach is based on an earlier theory or model [14]. Therefore, a directed content analysis method was used to determine and develop chronically-ill peoples’ self-efficacy as one of the concepts of FCEM.

### 2.2. Participants

The participants of the present study were 16 chronic ill patients (Rheumatism (*n* = 1), Myocardial infarction (*n* = 2), Multiple sclerosis(*n* = 1), Hypertension (*n* = 1), Diabetes mellitus (*n* = 3), Chronic renal failure (*n* = 4), Chronic obstructive pulmonary disease (COPD) (*n* = 1), Asthma (*n* = 2), and Angina (*n* = 1)) (key participants (nurses, and family caregivers (general participants). Primary (key) participants were chronically ill patients who were selected purposively and with maximum variation with respect to age, education and employment status, and the underlying chronic disease. Inclusion criteria were affliction with a chronic disease for at least three months, agreement to participate, and ability to verbally communicate, share information, and express feelings.

In line with guidance provided by the participants, caregivers (family members) and nurses were interviewed.

### 2.3. Data Collection

The qualitative component of the study was conducted from May 2015 to March 2016 in the internal medicine, neurological, and dialysis care wards of medical university hospitals in Iran. The first and second authors in a private room conducted 22 semi-structured interviews. These interviews started with open-ended questions such as “how are you today?” “How many days have you been admitted?” Demographic questions were posed, and exploratory and probing questions, such as “Can you explain more?” “What does it mean?”, were asked. The interviews were continued until data saturation was reached, meaning that no new findings were added. Based on the directed content analysis method, the questions were derived from the FCEM self-efficacy concept attribution, such as “What skills and abilities do you have for managing the problems associated with your chronic disease?” and “Please explain about your adherence to your treatment/dietary regimens”.

### 2.4. Data Analysis

Existing theory or research can provide predictions about the relationships among variables, thus helping to determine the initial coding scheme or relationships between codes. A qualitative directed content analysis approach has three main phases, preparation, organization, and reporting [12]. The preparation phase starts with selecting the unit of analysis, deciding what to analyze, and selecting a unit of meaning. Next, in the analytic process, the researcher strives to make sense of the data and to learn ‘what is going on’ and obtain a sense of the whole. In the organizing phase, the next step is to develop a categorization matrix and code the data according to the categories of the model. In the reporting phase, the researcher generates a link between the results and the data. The researcher describes the analysis process in as much detail as possible in appendices and tables.

After the interviews were recorded and transcribed verbatim, the researcher studied the text carefully in order to identify, based on the data in the text, concepts and patterns. The content analysis was started for interviews. The preparation phase started with selecting the unit of analysis containing the whole interview. Graneheim and Lundman pointed out that the most suitable unit of analysis is whole interviews that are large enough to be considered as a whole [15]. Before starting the encoding, the whole text was read several times, so that the researcher fully understood the data, was immersed in the data and got a comprehensive sense of the whole.

The organizing phase includes coding, creating categories, and abstraction [12]. After a categorization matrix is developed, all the data are reviewed and coded for correspondence with or exemplification of the identified characters of the category. We used open coding, then the lists of categories were grouped under higher order headings and abstraction was performed. Abstraction means formulating a general description of the research topic through generating categories [12]. In the reporting phase, appendices and tables were used to demonstrate the links between the data and the results.

To ensure accuracy and reliability of qualitative data, the scientific rigor criteria of Guba [16] and Lincoln [17] were used including credibility, dependability, transferability, and confirmability. Methods of determining credibil­ity were used, including review by participants and examination of data by expert colleagues. All researchers were engaged in the process of analyzing and synthesizing the data. Reliability was en­hanced through constant comparative analysis, return to the field (site), and constant ongoing communication with participants and presence in the field. For proportionality, the maximum variation sampling technique (gender, educational status, age, employment, and type of underlying condition) was used. Also, conformability of findings was assessed by an auditor familiar with qualitative re­search. To this end, parts of interview texts, together with relevant codes and categories that emerged, were examined and confirmed by two experts familiar with qualitative research. For dependability of research, the researcher accurately recorded and reported stages and processes of the study in sufficient detail, so that others could follow-up the research.

### 2.5. Ethical Considerations

This study was approved by the institutional review board (IRB), Research Council and Ethics Committee of the University (ID IR.TMU.REC.1394.79). The necessary permissions from the authorities of the study setting and the Ethics Committee of the Medical University to collect data and informed consent for the audiotaping were obtained from the participants. Moreover, they retained the absolute right to withdraw from the study voluntarily. We also guaranteed the confidentiality of their data.

## 3. Results

Most participants were female (58.0%), with a mean age of 49.50 years, range 29–64. Moreover, the mean of the interview length was 40.05 minutes. The descriptive data of the participants is presented in Table 1. The final analysis yielded 247 units of analysis dispersed in eight subcategories belonging to four generic-categories (Table 2).

### 3.1. The Cognitive Generic-Category

Cognitive self-efficacy is the ability of the chronic patient to develop their knowledge and understanding in relation to illness and self-care through individuals and scientific and virtual resources. The two subcategories of this generic category are developing health-related knowledge through other individuals (such as health workers and other patients) and developing health-related knowledge through the sources of scientific data (such as books, associations, the media, and the internet). These generic categories denote that patients who have cognitive self-efficacy can develop their health-related knowledge and obtain information about their illness, symptoms, treatments, and healthy lifestyle through referring to physicians, nurses, other patients, books, patient associations, the media, and the internet.

#### 3.1.1. Developing Health-Related Knowledge through Other Individuals

In order to obtain information about their self-care, treatment, illness, symptoms, complications, treatments, and so on, they pose questions to healthcare workers and patients who suffer from the same health conditions. In this way they learn how to care for themselves, obtain information about health and dietary regimen, and develop their health-related knowledge.

Which equipment is needed? Do they provide me with the necessary equipment? Can I take the equipment to my home?” (P. 9).

*I asked other patients about my dietary regimen,* i.e., *about foods that I can and cannot eat (P. 4).*

#### 3.1.2. Developing Health-Related Knowledge through the Sources of Scientific Data

Another strategy used by patients with cognitive self-efficacy which provides knowledge about welfare methods and health-related questions, is referring to sources of scientific data such as scientific texts, patient associations, the media, and the internet. These associations, by providing books and holding meetings or congresses; the internet, by gathering information about health and caring; and the media, by broadcasting a wide variety of health-related programs and providing people with the opportunity to take part in these directly and indirectly, provide the facilities to participate in the development of knowledge.

I asked the physicians of our association, about dietary regimen and foods that help my treatment (P. 3).

I search the internet. Moreover, I attempt to go forward, not backward through obtaining information from books and other sources (P. 3).

### 3.2. The Psycho-Emotional Generic Category

Self-efficacy is about one’s belief in his/her coping ability in specific situations. It affects thinking, behavioral, and emotional patterns at different levels of human experiences. It can control personal emotions and their management, and improve individuals’ psychological well-being and mental health. Psycho-emotional self-efficacy means that the patient will continue to live with illness by managing his excitement. Self-efficient individuals have positive attitudes toward life and are psycho-emotionally able to cope with difficult situations, changes, and stressors. The subcategories of this main category were living with the illness, and management of emotions.

#### 3.2.1. Living with the Illness

After achieving recovery from the acute phase of their illnesses, self-efficient patients are able to accept their illnesses, change their behaviors, show adaptive behaviors (such as close adherence to treatments), and continue their lives. Patients with self-efficacy attempt to maintain their morale, control their immediate situations, and tolerate disruptions in order to accept and cope with their illness to achieve fast recovery and continue their lives. After accepting the fact that they are really ill, self-efficient patients take responsibility for their illnesses and health, then show behaviors such as taking their medication everywhere and every time, procuring their medications before finishing their supply, and using their medications at the determined time.

*I need to adjust my life to this program because these are my medication and treatments (P. 12).*

It was here for the first time that Dr. F noticed and diagnosed my diabetes. At first, I did not accept it. Then, I returned to my hometown. There, doctors confirmed that I have diabetes. Thereafter, I gradually started to consider that I really have diabetes even though I couldn’t accept it. After that, I gradually accepted that the level of my blood sugar could be high. Currently, I say and accept that I’m diabetic (P. 12).

#### 3.2.2. Management of Emotions

Emotions such as fear, anxiety, tension, anger, and depression cause patients to underestimate their abilities. In other words, such emotions reduce their self-efficacy. Self-efficient patients control their illnesses, consequences, and complications, with the ability to identify factors that worsen their conditions, such as negative emotions, anger, crying, and depression. With decision making for controlling their anger and stressors, complications such as fatigue, anger, and pain can be prevented. Making an attempt to decide on and manage their emotions and stressors that reduce their quality of life and slow the processes of treatment, they improve their lives by employing strategies such as watching television.

I have decided here to take my medications regularly and avoid high levels of stress after my hospital discharge (P. 12).

They used to tell me that nervousness, rather than sugar overuse, increases my blood sugar. Therefore, I attempt to keep calm. Currently, I care for myself, keep my cool, and cry less frequently (P. 11).

### 3.3. The Functional Generic Category

Functional self-efficacy means that the chronically ill people can plan their lifestyle and self-care with trust in perception of his/her own abilities to perform daily and self-care activities, and also plan for his/her life so much so that he/she can maintain these abilities and positively affect others. The subcategories of this generic category included planning for life and performing self-care activities.

#### 3.3.1. Planning for Life

Patients with self-efficacy gradually understand that without thoughtful planning, life cannot be continued. Consequently, they plan house tasks alongside their daily rest and activities, avoid doing heavy tasks or those that take a long time, balance fluid intake, avoid high-fat or high-salt foods, use nutrients, and avoid unhealthy foods. Despite being affected by their chronic illnesses and problems, they perform their parenting responsibilities and take care of their children and families.

Currently, I cannot walk normally and get tired rapidly. Nonetheless, as I have planned for my life, I wake up in the morning at a predetermined time and go on throughout the day based on the plan. Thus, I can manage my life effectively (P. 3).

#### 3.3.2. Performing Self-Care Activities

One of the most significant components of chronic illness management is self-care. Self-care includes the learned and deliberate actions that people perform for themselves and their families in order to stay healthy, protect their physical and mental health, fulfill their psychosocial needs, and maintain their post-illness health. This health care can be adherence to treatments and taking care of one’s own body or one’s own psyche. In this study, participants showed that they overcome their problems by undertaking regular diagnostic tests, taking prescribed medications, and adjusting their medication based on their conditions, regular assessment of symptoms, regular visits to the doctor, and taking care of their own psyche by living happily.

I visit my doctor every two months. He prescribes some medication and I buy and take that (P.3).

I’ve noticed that in order to prevent convulsions, I should avoid going to bereavement ceremonies. I also don’t listen to sad music and keep myself in a happy environment (P. 3).

### 3.4. The Social Generic-Category

Social self-efficacy is one’s perceptions about his/her own ability to achieve good professional performance and adjust interpersonal relationships. This means that performing well in their jobs enables them to regulate their communication with others. The participants also confirmed good professional performance and adjustment of interpersonal relationships as instances of social self-efficacy.

#### 3.4.1. Professional Performance

Some chronically-ill patients lose or give up their employment due to the complications and problems of their chronic illnesses. However chronically-ill patients who are self-efficient attempt to adjust their work-related affairs through reducing the number of working hours or engaging in light work in order to be able to cope with their illnesses. Empowered and self-efficient individuals are able to effectively perform their work-related activities and adjust their work to their conditions.

I just supervise the work of my sons and teach them how to do it. Besides, I do activities related to my beehives. Moreover, I do the shopping and our family’s administrative affairs (P. 3).

#### 3.4.2. Adjusting Interpersonal Relationships

Relationships are the first prerequisite for social life. Strong and healthy interpersonal relationships promote resilience to problems and decrease vulnerability. Self-efficient patients can limit their relationships when a relationship bothers them or aggravates their illnesses and expand their relationships with those who boosted their morale or supported them.

I limit my relationships with those who bother me. In other words, I manage to visit them less frequently (P. 3). I attempt to have relationships with those who give me positive energy (P. 3).

## 4. Discussion

The aim of this study was to understand and develop patient’s self-efficacy. Self-efficacy is a multi-structural concept. It has been defined with terms such as the empowerment index, predisposing factors and acquired skills for the empowerment process. According to the findings, self-efficacy is the ability of patients to develop their knowledge and understanding in relation to illness and self-care through virtual and non-virtual personal and scientific resources, manage their excitement in a way that allows them to continue to live with their illness and self-care, plan for their lifestyle, have proper job performance, and have regular relationships with others. They are named as ‘cognitive’, ‘psycho-emotional’, ‘functional’, and ‘social’ dimenisions. The experience of chronically-ill people shows that self-efficacy is a multidimensional concept, the dimensions of which interact with each other, as found in the studies by Tahmassian [18].

The participants used the four dimensions of self-efficacy in order to resolve their illness-related problems, acquire adequate knowledge about their illnesses and treatments, control and manage the psycho-emotional processes of their lives, and establish good interpersonal relationships.

Empowerment appears in many different contexts, always as a cognitive process, that of enhancing the capacity of individuals or groups to make choices and to transform those choices into desired actions and outcomes [19]. As noted in this study, individuals attempt to develop their health-related knowledge through employing different strategies to be able to select the most appropriate treatment and dietary regimens, as noted by Mola [20].

In other words, self-efficacy is the link between knowledge and action, that leads to empowerment [7]. This is similar to the findings of the present study in that the participants attempted to develop their knowledge about their illnesses, symptoms, complications, and treatments, through referring to patient associations, reading books, asking others as well as healthcare workers, and seeking information from print and audiovisual medias. This is supported by Haghani’s report that self-efficacy can help individuals develop their knowledge and skills and use them in their daily practice [21].

The study findings revealed that another dimension of self-efficacy was the psycho-emotional dimension. The participants attempted to manage their stress and emotions, control their anger and accept their illnesses, modify behaviors, accept treatments, improve physical and mental health, and make wise decisions and, thus, feel greater levels of empowerment. As noted by Royani and Jamali individuals who have high self-efficacy are able to improve their stress management, decision-making and adopt more positive attitudes [22,23].

Another of these behaviors is coping. Participants strived to cope with their illnesses and situations, through a self-efficacy component, belief, that can affect different aspects of life through exerting control on life. For instance, controlling the immediate situation in the face of stressful events is a significant factor behind successful adaptation with the associated problems as observed in previous studies [24].

Our participants, despite various barriers, had also accepted responsibility toward their illnesses and attempted to manage their stress with great perseverance. They can cope with despair and failures, moreover, they consider a shortage as a temporary retreat rather than a final outcome, and inhibit stressors before they happen, as found in the study by Rezazadeh [25].

The patient with one or more chronic conditions is the real master of his own health and well-being. They decide about their lifestyles, physical activity, diet, taking medicines, and integrate external information with their attitudes, culture, and expectations [20].

Another dimension of self-efficacy is functional. Our self-efficient participants were able to perform their self-care activities. Basically, self-efficacy is defined by expressions such as “the factor contributing to or the indicator of the process of empowerment”. Therefore, patients who have a high level of self-efficacy are more confident about being empowered for doing self-care activities [22]. According to the studies by Sadegi, improved self-efficacy helps modify health-related behaviors and affects the levels of attempts, performance, and engagement in healthy behaviors such as symptom management, physical activity, healthy eating, treatment adherence, rest, and activity alternation. Consequently, this increases the functional capacity, in accordance with the findings of this study [26].

The fourth dimension of self-efficacy is the social dimension. Self-efficacy appears to be a better predictor of work performance than job satisfaction [27], while chronic illnesses can negatively affect individuals’ quality of life and employment, self-efficacy improves their professional performance and quality of life [28], this is similar to the findings from this study.

Relationships can be both facilitators and inhibitors of empowerment, and patients’ skills play an important role in managing these relationships [29]. That is similar to the findings of this study; patients limited their unhealthy relationships and expanded their healthy relationships and were satisfied with living with their family members.

Such relationships make life enjoyable and enable individuals to effectively cope with long-term strains [30]. Participants acknowledged that communicating with members of their nuclear and extended family (according to Iranian society), controlling these communications, and deciding on the development or limitation of them helps to meet some of the needs for daily activity and improves self-efficacy.

In the current study, the dimensions of self-efficacy have been presented based on real experiments, background, and the family-centered empowerment model. So, given that chronic patients are always at risk of illness, it is better to use self-efficacy as a strategy to achieve their empowerment and improve their health.

## 5. Limitations 

The most important limitations of the present study were the small sample size of participants, unequal numbers of different patients, and difficulty in access to participants. 

## 6. Conclusions

The results of the present study indicate that self-efficacy of chronic people consists of cognitive, psycho-emotional, functional, and social dimensions that can be used to assess empowerment. Moreover, identification of dimensions helps holistic assessment of patients’ self-efficacy. These findings can be a baseline for future research in developing effective health interventions by nursing for increasing self-efficacy and empowerment in people with chronic diseases. This can result in the development of chronically ill patients’ personal knowledge, improving their emotions and stress management, strengthening their social relationships, and boosting their caring performance.

## Figures and Tables

**Table 1:** Participants’ characteristics.

Number	Age	Gender	Type of Disease	Number of Interviews	Interview Length (Minutes)
1	29	Male	Rheumatism	2	49
2	58	Male	Chronic obstructive pulmonary disease	1	45
3	37	Male	Diabetes mellitus	1	45
4	49	Female	Diabetes mellitus	2	40
5	64	Female	Asthma	1	39
6	38	Male	Asthma	2	40
7	53	Female	Myocardial infarction	1	35
8	71	Male	Hypertension	1	35
9	65	Female	Angina	1	32
10	57	Female	Chronic renal failure	1	35
11	41	Male	Chronic renal failure	1	40
12	34	Female	Multiple sclerosis	1	51
13	72	Female	Chronic renal failure	1	20
14	56	Male	Diabetes mellitus	1	18
15	36	Male	Myocardial infarction	1	25
16	42	Female	Chronic renal failure	1	32

**Table 2:** The main categories, generic categories, and subcategories of the study.

Main Categories	Generic Categories	Subcategories
Self-efficacy	Cognitive	Developing health-related knowledge through other individuals
		Developing health-related knowledge through the sources of scientific data
	The psycho-emotional	Living with the illness
		Management of emotions
	The functional	Planning for life
		Performing self-care activities
	The social	Professional performance
		Adjusting interpersonal relationships
